# Retraction: Mechanisms of adipocyte regulation: Insights from *HADHB* gene modulation

**DOI:** 10.1371/journal.pone.0353719

**Published:** 2026-07-14

**Authors:** The PLOS One Editors

Following the publication of this article [1], concerns have been raised about Fig 1. Specifically:

- The Fig 1C Control panel of this article [1] appears similar to the Fig 3E panel of [2] when rotated, despite being used to represent different experimental conditions.
- When rotated, the Fig 1C si-HADHB panel in [1] appears similar to the left panel of Fig 6A in [3] and the Fig 5B Ad-GFP 0d panel in [4], despite being used to represent different experimental conditions.

The authors did not respond to the journal’s queries about these concerns. In light of the above unresolved concerns that question the reliability and integrity of the reported results, the *PLOS One* Editors retract this article.

All authors either did not respond directly or could not be reached.
